# Exercise Regulates Mitochondrial Quality Control: Maintenance and Remodeling of Skeletal Muscle Homeostasis

**DOI:** 10.3390/biology15151240

**Published:** 2026-07-27

**Authors:** Huiying Zhao, Min Chen

**Affiliations:** 1 School of Exercise and Health, Shanghai University of Sports, Shanghai 200438, China; zhaohy1237@163.com; 2 School of Physical Education, Shanghai University of Sports, Shanghai 200438, China

**Keywords:** mitochondria, mitochondrial quality control, skeletal muscle, exercise

## Abstract

Skeletal muscle, as the largest metabolic organ, maintains its homeostasis highly dependent on the precise regulation of mitochondrial quality control. Mitochondrial quality control (MQC) encompasses three core aspects: mitochondrial biogenesis, dynamic balance, and autophagy. All of these jointly ensure mitochondrial network functional integrity. MQC imbalance is mainly manifested as decreased mitochondrial biosynthesis capacity, disordered fusion and division dynamics, and reduced autophagy clearance efficiency. MQC imbalance can lead to atrophy of skeletal muscles, metabolic dysfunction, and decline in motor function. As a physiological stress stimulus, exercise can precisely regulate MQC through multiple targets and pathways and restore the homeostasis of skeletal muscles. Exercise activates AMPK-PGC-1α to promote mitochondrial biogenesis, regulates MFN1/2, OPA1, and DRP1 to optimize mitochondrial dynamics, and activates the PINK1/Parkin pathway and receptor-mediated autophagy pathway to enhance mitochondrial autophagy. The regulatory effects of different exercise modes on MQC vary significantly. Aerobic exercise focuses on promoting mitochondrial biogenesis and fusion, while high-intensity interval training can more efficiently activate the autophagy pathway. Resistance exercise, on the other hand, requires a longer period to manifest its regulation of dynamic proteins. This article systematically reviews the molecular regulatory mechanism of MQC and its impact on skeletal muscle imbalance and elaborates on the mechanisms by which exercise regulates the remodeling of skeletal muscle through MQC. This article also further compares the differential effects of different exercise modes on the regulation of mitochondrial quality control to maintain skeletal muscle homeostasis. Future research needs to further explore the dose and effect relationship of exercise on regulating MQC and the optimal combination of exercise modes to provide a scientific basis for formulating precise and safe exercise intervention strategies.

## Simple Summary

Skeletal muscle, the body’s largest metabolic organ, relies critically on mitochondrial integrity for proper function. Mitochondrial quality control encompasses biogenesis, repair, and clearance mechanisms that preserve organelle homeostasis. Dysfunction in these pathways, arising from aging, malnutrition, or disease, precipitates muscle atrophy, impaired bioenergetics, and metabolic dysfunction. This review elucidates the molecular mechanisms governing mitochondrial quality control in skeletal muscle and examines the consequences of its failure. Notably, exercise emerges as a potent, cost-effective intervention to restore mitochondrial homeostasis. Strategic exercise prescription represents a promising approach to sustain musculoskeletal health throughout the lifespan, warranting further investigation into optimal dosing for diverse populations.

## 1. Introduction

Skeletal muscle is the largest metabolic organ in the human body, accounting for approximately 40% of body weight. It plays an irreplaceable role in maintaining the body’s motor functions, energy metabolism, and overall homeostasis [1]. The quality of mitochondria, as the core cellular organelle for energy supply in skeletal muscles, directly determines the state of muscle fibers [2]. Through oxidative phosphorylation, mitochondria generate Adenosine Triphosphate (ATP) to power muscle contraction and engage in calcium homeostasis, reactive oxygen species (ROS) production, and apoptosis. Abnormal mitochondrial function causes energy metabolism disorders in muscle fibers, resulting in pathological changes such as muscle weakness, fatigue, and atrophy. Mitochondrial quality control (MQC) is defined as a coordinated network of interconnected processes, including mitochondrial biogenesis, dynamics (fusion and fission), and autophagy, which collectively maintain mitochondrial structure, functional integrity, and proteostasis, thereby ensuring cellular energy homeostasiss [3]. “Mitochondrial quality” therefore does not merely denote mitochondrial quantity or density but rather reflects the integrated functional status of the organellar network, encompassing respiratory capacity, redox homeostasis, membrane potential integrity, and the efficient turnover of damaged components [4]. Aging, unhealthy lifestyles and various pathological conditions can all lead to MQC imbalance, manifested as decreased mitochondrial biosynthesis capacity, unbalanced mitochondrial dynamics due to fusion and division disorders, and reduced autophagy clearance efficiency. Eventually, this leads to skeletal muscle atrophy, metabolic dysfunction and decline in motor function [5]. As a physiological stress stimulus, exercise has been proven to precisely regulate MQC through multiple targets and pathways, including promoting mitochondrial biogenesis, optimizing mitochondrial dynamics, and enhancing mitochondrial autophagy, thereby playing a crucial role in maintaining the homeostasis of skeletal muscles [6]. However, the regulatory effects of different exercise patterns on each aspect of MQC vary significantly, and the molecular mechanisms involved are also different. In this review, we systematically reviewed the molecular regulatory mechanisms of mitochondrial biogenesis, dynamics, and autophagy and elaborated on the impact of MQC imbalance on the homeostasis of skeletal muscle. We summarized the mechanisms by which exercise regulates mitochondrial quality control to reshape skeletal muscle and compared the differential regulatory effects of different exercises on MQC, aiming to provide a more theoretical basis for optimizing exercise prescriptions and optimizing mitochondrial quality control to improve skeletal muscle health.

## 2. MQC and Skeletal Muscle Homeostasis

### 2.1. Mitochondrial Biogenesis

Mitochondrial biogenesis denotes the process whereby new healthy mitochondria are generated through the coordinated action of nuclear and mitochondrial genes, replacing damaged organelles to sustain cellular energy supply and functional homeostasis [7]. Peroxisome proliferator-activated receptor γ coactivator-1 (PGC-1), which belongs to nuclear receptor superfamily, is regarded as the most important regulator of mitochondrial biogenesis [8]. PGC-1 serves as a key link between nutrients and energy metabolism. It can influence skeletal muscle metabolism and fats by regulating mitochondrial oxidative phosphorylation [9]. The PGC-1 family consists of Peroxisome proliferator-activated receptor γ coactivator α (PGC-1α), Peroxisome proliferator activated receptor γ coactivator 1β (PGC-1β), and PGC-1-related coactivator (PRC). PGC-1α, as a sensor for nutritional signaling molecules, is important in energy metabolism processes [10]. PGC-1β specifically regulates lipid metabolism and adipocyte differentiation [11]. PRC primarily functions in mitochondrial biogenesis and cell proliferation. Normal muscle fiber contraction involves ATP into adenosine diphosphate (ADP) and adenosine monophosphate (AMP). The phosphorylated AMP-activated protein kinase factor (AMPK) then acts on SIRT1, thereby activating the deacetylation of genes related to energy metabolism [12]. In addition to AMPK, calcium/calmodulin-dependent protein kinases (CaMKs) constitute another major pathway controlling mitochondrial biogenesis. Skeletal-muscle-specific overexpression of a constitutively active form of CaMKIV in transgenic mice augments mitochondrial DNA replication, upregulates PGC-1α expression, enhances fatty acid oxidation enzymes, and reduces fatigue susceptibility [13]. CaMKII is acutely activated by exercise-induced Ca^2+^ release from the sarcoplasmic reticulum and synergizes with AMPK to phosphorylate class IIa histone deacetylases (HDACs), relieving their repression on MEF2-driven PGC-1α transcription [14]. Furthermore, p38γ is activated by mechanical stress during contraction and drives PGC-1α expression through phosphorylation of activating transcription factor-2 and MEF2 [15]. Therefore, the activation of PGC-1α-dependent mitochondrial biogenesis during exercise integrates AMPK, CaMK, and p38 MAPK pathways. PGC-1- and ERR-induced regulator in muscle 1 is a muscle-specific regulator of mitochondrial bioenergetics and oxidative capacity, originally identified as a downstream effector of the PGC-1α/ERRα axis [16]. PERM1 is predominantly expressed in skeletal muscle and associates with CaMKII, regulating its activation and thereby enhancing MEF2-mediated PGC-1α transcription [17]. PERM1 gain-of-function in murine skeletal muscle increases mtDNA content, oxidative phosphorylation protein expression, and fatigue resistance, while PERM1 knockdown attenuates exercise-induced mitochondrial biogenesis and CaMKII/p38 MAPK activation [18]. Overexpression of PGC-1α can activate downstream target genes such as nuclear respiratory factor NRF-1/2 (NRF-1/2) and estrogen-related receptors, promoting mitochondrial DNA replication, the synthesis of respiratory chain subunits, and the enhancement of fatty acid oxidation capacity, increasing mitochondrial content and oxidative metabolic capacity [19]. Mitochondrial biogenesis requires the coordinated expression of two distinct genomes: the nuclear genome, which encodes the vast majority of mitochondrial proteins including PGC-1α, NRF-1/2, and TFAM, and the mitochondrial genome (mtDNA), which encodes 13 essential subunits of the electron transport chain (ETC) [20]. The nuclear respiratory factors NRF-1 and NRF-2 serve as critical nodes that link nuclear gene expression to mtDNA transcription [21]. Upon activation by PGC-1α, NRF-1/2 translocate to the nucleus and drive the expression of nuclear-encoded ETC subunits, mitochondrial import machinery, and TFAM [22]. TFAM is subsequently imported into mitochondria, where it binds to mtDNA, compacts it into nucleoids, and activates mitochondrial transcription and replication, thus completing the nucleo-mitochondrial regulatory loop [23].

### 2.2. Mitochondrial Dynamics

Mitochondrial dynamics refers to mitochondrial division and fusion within the cell [24]. Mitochondria can fuse and connect with each other to form a network-like structure, or they can split and exist as separate entities [25]. Mitochondrial fission produces small and fragmented mitochondria, which is beneficial for mitochondrial transport, damaged mitochondria separation, and mitochondria distribution to daughter cells during cell division [26]. Fission allows cells to more effectively respond to fluctuating energy demands and dispose of damaged mitochondria when required [27]. Mitochondrial fusion enables content exchange between different mitochondria and helps prevent mitochondria degradation, thereby sustaining normal physiological functions [28]. Skeletal muscle is a tissue that highly relies on energy supply. The dynamic balance of the mitochondrial network is of vital importance for the contraction, repair and metabolic regulation of muscle fibers [29]. When the fusion process intensifies, mitochondria form a highly interconnected network structure, which is conducive to the enhancement of oxidative phosphorylation capacity and is in line with the functional characteristics of slow muscle fibers. When the fission process predominates, it will promote glycolytic metabolism, which is in line with the functional characteristics of fast muscle fibers [30]. During muscle atrophy, whether it is age-related sarcopenia or disuse-induced muscle atrophy, it has been observed that the mitochondrial network becomes more severe, and fusion–fission balance is disrupted [31]. Excessive mitochondrial fission leads to more fragmented mitochondria, which not only reduces the efficiency of energy production but also triggers oxidative stress and apoptosis signals, accelerating the degeneration of muscle fibers [32]. The fusion disorder will impede the exchange of mitochondrial contents, causing damaged mitochondrial DNA and misfolded proteins and further damaging the mitochondrial quality control [33].

### 2.3. Mitochondrial Autophagy

Mitochondrial autophagy is a selective autophagy process that ensures the clearance of damaged mitochondria while promoting the generation of normal mitochondria [34]. PTEN-induced kinase 1 (PINK1) is a serine/threonine protein kinase located on the outer mitochondrial membrane. The Parkin protein is an E3 ubiquitin protein ligase encoded by the Parkin gene. Mitochondrial autophagy mainly involves two pathways: the PINK-1/Parkin-dependent autophagy pathway and the receptor-mediated autophagy pathway. In the PINK-1/Parkin-dependent pathway, the changes in mitochondrial membrane potential play a crucial role as a sensor [35]. When mitochondria are in a healthy state, their membrane potential remains at a relatively high level. At this time, the PINK1 protein can be normally imported into the inner mitochondrial membrane and rapidly degraded. There is little accumulation of PINK1 on the outer membrane [36]. However, when mitochondria are stimulated by ROS or other external factors causing a decrease in membrane potential, the import of PINK1 is blocked, and it accumulates in large quantities on the outer membrane of the mitochondria [37]. Accumulated PINK1 activates itself via phosphorylation and then recruits Parkin to damaged mitochondria [38]. Upon Parkin activation, it catalyzes the ubiquitination modification of outer mitochondrial membrane proteins [39]. Optineurin (OPTN), CALCOCO2 (NDP52), and Sequestosome-1 (P62) recognize these ubiquitination marks through their ubiquitin-binding domains. At the same time, they utilize the LC3-interacting region (LIR) to bind to the Microtubule-associated protein 1 light chain 3 (LC3) protein on the autophagosome, forming mitochondrial autophagosomes. Eventually, they merge with lysosomes to finish degradation [40]. It is worth noting that PINK1 can also directly phosphorylate ubiquitin molecules to directly recruit OPTN and NDP52, transporting the damaged mitochondria into lysosomes for eventual degradation [41]. Receptor-mediated autophagy mainly relies on specific outer mitochondrial membrane receptor proteins and is usually triggered by starvation or hypoxia. FUN14 domain-containing protein 1 (FUNDC1), Bcl-2/adenovirus E1B 19-kDa interacting protein 3 (BNIP3), and its homolog NIP3-like protein X (NIX), as typical hypoxia-induced proteins, can directly interact with LC3 and initiate mitophagy without ubiquitination marking [42]. These receptor proteins not only can anchor the autophagosome membrane structure through their LIR motifs but also can regulate mitochondrial dynamics and promote mitochondrial fission to facilitate autophagic degradation [43]. Furthermore, studies have shown that Parkin can ubiquitinate NIX and BNIP3, and BNIP3 can also interact with the mitochondrial-localized PINK1, thereby enhancing Parkin-mediated mitochondrial autophagy. These two pathways exhibit cross-regulation and synergy [44]. Satellite cells, as the reserve precursor cells of skeletal muscle, are located between the basal lamina and sarcolemma of muscle fibers and remain in an undifferentiated and quiescent state. The remodeling of the mitochondrial network plays a crucial role in the activation and regenerative capacity of satellite cells. The loss of mitochondrial division in satellite cells leads to disordered electron transport chains, reduced efficiency of oxidative phosphorylation metabolism, and impaired mitochondrial autophagy, all of which collectively result in decreased satellite cell proliferation and failure of regeneration [45]. During the differentiation and maturation process of myoblasts, they further adjust their metabolic characteristics through the division of mitochondria and autophagy [46]. Ultimately, these differentiated myoblasts fuse with the damaged muscle fibers, restoring the structure and function of the muscle fibers, or combine to form myotubes, which subsequently mature into new skeletal muscle fibers.

## 3. Impact of MQC Imbalance on Skeletal Muscles

MQC imbalance is influenced by multiple factors, including aging, iron homeostasis disorder, energy metabolism disorder, and dysfunction of the autophagy–lysosome system. These factors interweave with each other, jointly disrupting the biogenesis, dynamic balance, and autophagic clearance of mitochondria, ultimately leading to skeletal muscle damage.

### 3.1. Quality Deterioration

The regulation of mitochondrial biogenesis and energy metabolism critically depends on PGC-1α [47]. PGC-1α assists in activating multiple nuclear receptors through ligand-dependent mechanisms, regulating key physiological processes such as gluconeogenesis, glucose transport, fatty acid oxidation, oxidative phosphorylation, and muscle fiber type transformation [48]. The decreased expression and oxidative capacity of PGC-1α is often accompanied by skeletal muscle atrophy [49]. In mice with high expression of PGC-1α, the mitochondrial biogenesis response is enhanced, type I and oxidized type IIa fibers increase significantly, and the fatigue resistance improves [50]. On the contrary, the absence of PGC-1α and PGC-1β in muscles would lead to a significant reduction in the expression of genes related to mitochondrial respiration, electron transport chain and oxidative phosphorylation, as well as the motor ability [51]. Kim et al. [52] used the PGC-1α inducer to screen out an NSAID, indomethacin. They conducted tests on aged mice, muscle atrophy models, and cell models, and found that the protein kinase B/ribosomal protein S6 kinase pathway and PGC-1α promoted muscle protein synthesis and inhibited protein degradation, thereby increasing the muscle mass of aged mice.

### 3.2. Metabolic Impairment

Mitochondrial dynamics imbalance is one of the core mechanisms underlying impaired metabolic function in skeletal muscles. The dynamic balance between mitochondrial fusion and fission is disrupted by excessive ROS and Ca^2+^ homeostasis disorders, and this further triggers mitochondrial autophagy and apoptosis processes [53]. The key proteins responsible for fusion mainly include Mitofusin 1 (MFN1), Mitofusin 2 (MFN2), and OPA1 mitochondrial dynamin-like GTPase (OPA1). Key proteins responsible for division mainly include Dynamin-related protein 1 (DRP1) and Mitochondrial Fission 1 Protein (FIS1). Abnormal expression of these proteins will lead to mitochondrial morphological and functional disorders. Despite their high homology, MFN1 and MFN2 possess distinct functional roles. The absence of MFN1 causes more severe mitochondrial fragmentation than that of MFN2 [54]. The inhibition of MFN2 expression will increase ROS and activate MAPK8/NF-κB, leading to insulin resistance and reduced glucose uptake [55]. OPA1 is mainly responsible for the formation and stability of inner mitochondrial membrane folds [56]. The mutation of OPA1 will disrupt fusion of the inner membrane, causing severe mitochondrial dysfunction, resulting in a decline in muscle mass and even premature death [57]. Excessive division is also harmful. Abnormal expression of DRP1 leads to impaired hydrogen peroxide production induced by ceramides and impaired energy metabolism. Dulac et al.’s experiments demonstrated that as muscle develops, DRP1 and phosphorylated proteins increase significantly [58]. Intramuscular injection of adenovirus into adult mouse skeletal muscles and knockdown of DRP1 resulted in severe muscle atrophy in the mice after 4 months. Forkhead box O3, a regulator of atrophy genes, increased, as did muscle atrophy-specific ubiquitin ligase 1, which is linked to muscle mass loss. In the differentiated muscle tubes of C2C12 cells with the DRP1 gene knocked out, the absence of DRP1 led to a significant reduction in the expression of genes encoding fast-contracting fibers [59]. Myosin heavy chain 1 and Myosin heavy chain 4 can be restored by exogenous re-expression of DRP1 in cells that have been knocked out for DRP1. This indicates that DRP1 is crucial for the expression of myosin heavy chain genes in fast muscle fibers. The deficiency of DRP1 causes the mitochondria to become larger in morphology and exhibit abnormal functions. These mitochondria signal the nucleus to activate the ubiquitin–proteasome system and unfolded protein response. Meanwhile, changes in mitochondrial volume increase mitochondrial Ca^2+^ uptake, leading to muscle fiber death [60]. FIS1 deficiency causes mitochondrial swelling and hyperfusion, leading to dysregulated mitochondrial autophagy. After reduction of FIS1, ubiquinated proteins accumulate in the muscles, and these proteins, by binding to LC3 proteins, induce abnormal mitochondrial autophagy and result in muscle atrophy and degeneration [61].

### 3.3. Abnormal Autophagy

**Figure 1:**
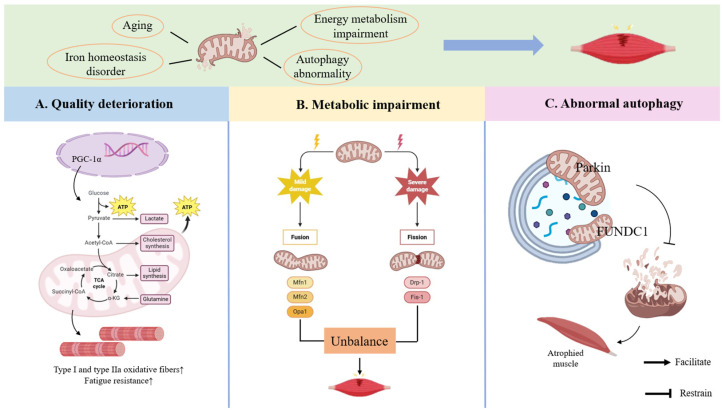
MQC imbalance affects skeletal muscle homeostasis through three interrelated pathways. (**A**) Weakened PGC-1α-mediated mitochondrial biogenesis reduces the generation of new functional mitochondria, thereby diminishing the capacity for mitochondrial renewal and contributing to an overall decline in mitochondrial network quality. (**B**) Aberrant expression of fusion and fission proteins causes dynamic imbalance, resulting in metabolic impairment. (**C**) Impaired mitophagy mediated by Parkin and FUNDC1 hinders the clearance of damaged mitochondria. These three defects interweave and collectively disrupt mitochondrial function, ultimately damaging skeletal muscle.

Mitochondrial autophagy is essential for the removal of damaged or senescent mitochondria and preservation of skeletal muscle homeostasis [62]. Suppressed mitophagy-induced mitochondrial dysfunction has been implicated as a contributing factor in certain types of skeletal muscle atrophy [63]. Parkin is indispensable in maintaining muscle contraction and mitochondrial functions [64]. Almost all types of skeletal muscle atrophy are accompanied by decreased Parkin or a reduction in mitochondria within the muscle cells. Impaired mitophagy can contribute to muscle wasting in disease-specific contexts, though the extent of its involvement may vary across different atrophy conditions. For instance, in chronic obstructive pulmonary disease (COPD)-related sarcopenia, reduced Parkin-mediated mitophagy was found to increase mitochondrial ROS, which subsequently activated muscle RING-finger protein-1 and promoted muscle atrophy [65]. Similarly, in a mouse model of sepsis, overexpression of Parkin attenuated mitochondrial dysfunction and alleviated sepsis-induced muscle fiber atrophy [66]. Apart from the PINK1/Parkin pathway, the receptor-dependent mitochondrial autophagy pathway is also crucial. The specific knockout of the activating molecule in Beclin 1-regulated autophagy protein 1 in mouse skeletal muscle can severely damage skeletal muscle quality and mitochondrial autophagy [67]. The regulation of muscle mitochondrial quality and the maintenance of metabolic homeostasis are also critically dependent on FUNDC1. FUNDC1 not only promotes autophagy but also participates in mitochondrial division and maintains the iron and protein homeostasis of mitochondria. Within mouse skeletal muscle tissue, the specific deletion of FUNDC1 leads to LC3-mediated mitochondrial defects, thereby impairing mitochondrial energy metabolism and reducing the utilization of muscle fat and endurance during exercise [68] (Figure 1).

## 4. Exercise Regulates Skeletal Muscles Homeostasis

MQC imbalance can have a serious impact on the homeostasis of skeletal muscles. Effective intervention methods to restore MQC and restore the function of skeletal muscles are particularly important. Exercise, as a physiological stimulus, has been proven to precisely regulate MQC through multiple targets and pathways, including promoting mitochondrial biogenesis, optimizing mitochondrial dynamics, and enhancing mitochondrial autophagy, thereby maintaining skeletal muscle homeostasis.

### 4.1. Promotion of the Biosynthesis of Mitochondria in Skeletal Muscle

Exercise activates multiple signaling pathways to promote mitochondrial biogenesis, mainly through balancing AMPK-PGC-1α axis activation and AMPK-mTORC1 sequential switching. During exercise, muscle contraction leads to a sharp increase in energy consumption, and the intracellular AMP/ATP ratio rises, thereby activating AMPK [69]. AMPK activation leads to direct PGC-1α phosphorylation, boosting its stability and transcriptional activity—a key step in starting mitochondrial biogenesis [70]. As a key regulatory factor, when PGC-1α is significantly increased, it binds to NRF1/NRF2, initiating the transcription of mitochondrial genes in the nucleus, thereby promoting mitochondrial DNA replication and transcription [71]. AMPK not only directly phosphorylates PGC-1α but also releases its inhibition on the PGC-1α pathway by phosphorylating Folliculin Interacting Protein 1 (FNIP1), thereby initiating mitochondrial generation. The nuclear receptor miR-499 feedback loop involving FNIP1 further strengthens this process. Activated AMPK can also inhibit the Ser79 site of the downstream target acetyl-CoA carboxylase (ACC), thereby inhibiting fatty acid synthesis and promoting fatty acid oxidation, responding rapidly to energy stress signals and promoting catabolic energy supply [72]. In addition, the mechanical load and calcium ion fluctuations caused by exercise can activate the Ca^2+^/calmodulin-dependent protein kinases (CaMK) pathway, which works in synergy with AMPK to activate PGC-1α. The p38 MAPK pathway provides an additional layer of regulation in exercise-induced mitochondrial biogenesis. Muscle-contraction-induced mechanical stress activates p38 MAPK, particularly the p38γ isoform, which phosphorylates and activates the transcription factors ATF-2 and MEF2 [73]. Phosphorylated ATF-2 binds to the cAMP response element on the PGC-1α promoter, while activated MEF2 binds to its cognate MEF2 binding sequence, together driving PGC-1α gene transcription [74]. In mice with muscle-specific deletion of p38γ, exercise-induced increases in PGC-1α mRNA and mitochondrial protein markers are markedly blunted, confirming the functional importance of p38γ in mitochondrial adaptation to exercise [75]. Exercise also promotes the Mitochondrial Open Reading Frame of the 12S rRNA-c (MOTS-c), and this mitochondrial hormone can reverse-activate the AMPK/PGC-1α pathway, enhancing mitochondrial respiratory function [76]. Nitric oxide (NO) has emerged as an additional modulator of mitochondrial biogenesis in skeletal muscle. Both endothelial NOS (eNOS) and neuronal NOS (nNOS) are expressed in skeletal muscle fibers and can be activated by exercise-induced shear stress and calcium influx [77]. NO donors upregulate PGC-1α mRNA and protein, increase NRF-1 and TFAM expression, and enhance mitochondrial respiration in cultured myotubes through an AMPK-dependent mechanism [78].

**Figure 2:**
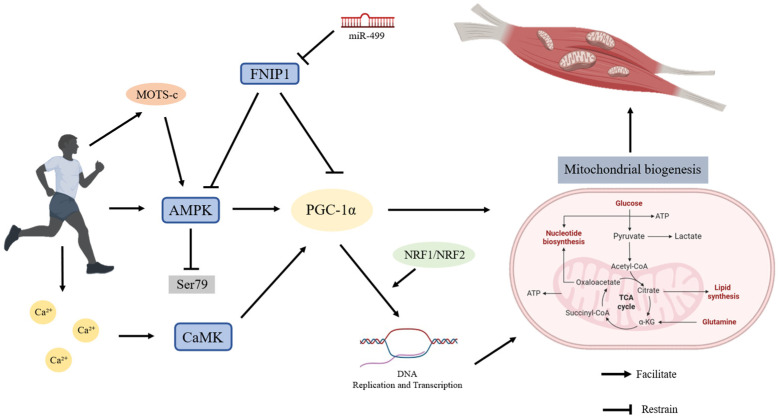
Exercise-triggered muscle contraction activates AMPK, which phosphorylates PGC-1α directly, boosting its stability and transcriptional activity. PGC-1α binds to NRF1/NRF2 to initiate nuclear transcription of mitochondrial genes and promote mitochondrial DNA replication. At the same time, AMPK phosphorylates FNIP1 to relieve its inhibition of the PGC-1α pathway. Mechanical load and calcium ion fluctuations caused by exercise can activate the CaMK pathway to synergistically activate PGC-1α. Mitochondrial-derived peptide MOTS-c can also promote PGC-1α by activating AMPK. Moreover, AMPK inhibition of mTORC1 promotes catabolism during exercise, and the recovery period after exercise, when mTORC1 activity rebounds, can drive protein synthesis, repair micro-damage, and maintain the assembly of mitochondrial enzyme complexes.

It is worth noting that the regulation of skeletal muscle homeostasis by exercise is not only dependent on the AMPK-PGC-1α axis, but more importantly, it relies on the precise temporal coordination between AMPK and the mTORC1 signaling pathway [79]. During the exercise, AMPK inhibits the activity of mTORC1 by phosphorylating TSC2 and Raptor, ensuring that energy is prioritized for muscle contraction and promoting catabolism [80]. During the recovery period after exercise, as the energy state returns to normal, the activity of mTORC1 gradually increases. It activates the downstream effectors S6K1 and 4E-BP1 through phosphorylation, initiating the translation process, driving protein synthesis, repairing exercise-induced micro-injuries, and maintaining the assembly of mitochondrial enzyme complexes [81]. A study conducted on non-endurance-trained men found that a 6 week endurance training program combined with pyrroloquinoline quinone supplementation significantly increased the level of the PGC-1α protein, indicating that exercise and nutritional intervention have a synergistic effect in promoting mitochondrial biogenesis [82]. At the epigenetic level, the AMPK activation induced by exercise can also regulate mRNA and protein levels of the AMPKα2 gene by influencing the methylation status of its promoter [83] (Figure 2).

### 4.2. Regulating Mitochondrial Dynamics to Reshape the Homeostasis of Skeletal Muscle

Mitochondrial fusion is a crucial process for maintaining the integrity and functionality of the mitochondrial network. It is carried out by outer membrane fusion proteins MFN1 and MFN2, as well as the inner membrane fusion protein OPA1 [69]. In mice with specific knockout of MFN1 and MFN2 in adult skeletal muscle, the locomotor ability showed a progressive decline, which was mainly attributed to the decreased activity of electron transport chain complexes I and IV as well as the reduced expression of corresponding subunits [84]. Exercise can significantly upregulate these proteins, thereby promoting the mitochondrial network. A study using a nematode model showed that regular exercise can increase AMPK. Under the action of AMPK, MFN2 also increases, promoting mitochondrial fusion and thereby improving skeletal muscle aging [85]. This result has also been confirmed in aging mice. Long-term exercise can enhance the mitochondrial fusion mediated by AMPK/MFN2 in aged mice and has an improving effect on age-related muscle atrophy [86]. OPA1 plays a central role in regulating the structure of mitochondrial cristae and the fusion of inner membranes. Its functional state directly affects the efficiency of the electron transport chain [87]. OPA1 exists in two forms: long and short. The ratio of these two forms is closely related to the adaptive response of mitochondrial stress. Exercise training can regulate the processing of OPA1, thereby optimizing mitochondrial function [88]. In a mouse model of Duchenne muscular dystrophy type 1, it was found that a single exercise session can stimulate the plasticity of skeletal muscle mitochondria, improve OPA1, and coordinate fusion and division signals [89].

**Figure 3:**
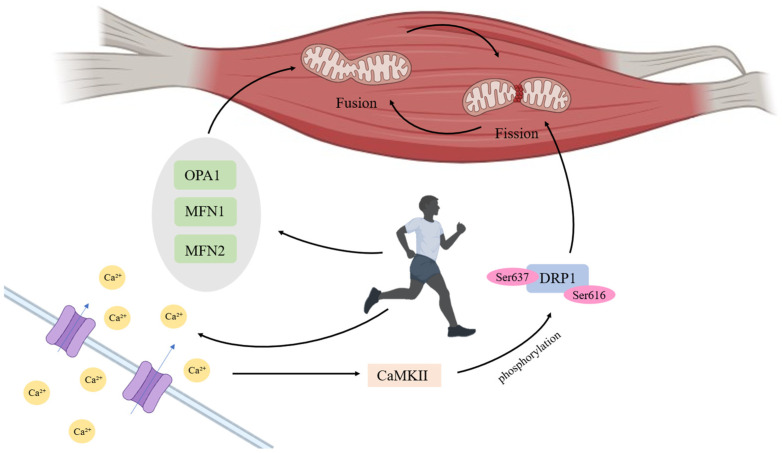
Exercise regulates mitochondrial dynamics to re-establish the homeostasis of skeletal muscles, manifested by promoting fusion and precisely controlling the coordinated mechanism of division. In terms of fusion, exercise significantly upregulates MFN1, MFN2, and OPA1, strengthening mitochondrial network integrity. In terms of division, acute exercise promotes division through CaMKII phosphorylation of DRP1 Ser616 to eliminate damaged mitochondria, while long-term training inhibits excessive division by reducing the phosphorylation ratio of DRP1 Ser616/Ser637. This dynamic equilibrium between fusion and fission allows the mitochondrial network to adapt to alterations in energy demands, thereby preserving the integrity and contractile function of muscle fibers.

DRP1 is a key protein that regulates mitochondrial division. Its activity is mainly regulated by phosphorylation at the serine 616 (Ser616) and serine 637 (Ser637) sites. Acute exercise can promote mitochondrial division by altering the phosphorylation state of DRP1, thereby maintaining the dynamic balance of the mitochondrial network in skeletal muscle. Specifically, the calcium ion influx induced by exercise can activate calcium/calmodulin-dependent protein kinase II (CaMKII). This kinase directly phosphorylates the Ser616 site of DRP1, accelerating the process of mitochondrial division and eliminating dysfunctional mitochondrial fragments [90]. This mechanism is particularly pronounced after acute exercise. The phosphorylation level of DRP1 Ser616 rises rapidly and gradually returns to a baseline level during the recovery period [91]. Excessive mitochondrial division leads to fragmentation of mitochondria, damaging their functions. During long-term training, exercise finely controls mitochondrial division activity by regulating DRP1 Ser616 and Ser637 phosphorylation. High-intensity interval training (HIIT) significantly lowers the DRP1 Ser616/Ser637 phosphorylation ratio in high-fat-diet-induced obese mice, inhibiting excessive mitochondrial division and improving mitochondrial quality [92]. At the same time, such movements can also upregulate MFN2 to prevent the collapse of the mitochondrial network, ensuring that the fragmented parts can be efficiently fused or recycled through the autophaHIF-αgy pathway [93]. This fission–fusion coordination is crucial for skeletal muscle mitochondrial homeostasis. The dynamic regulation induced by exercise makes the mitochondrial network more adaptable to changes in energy demands, reduces the release of apoptotic signals, and maintains the integrity and contraction function of muscle fibers (Figure 3).

### 4.3. Regulating Mitochondrial Autophagy to Maintain Skeletal Muscles Health

PINK1/Parkin is the central mechanism for mitophagy, crucially maintaining mitochondrial homeostasis in skeletal muscle. As a physiological stress, exercise can activate PINK1/Parkin by increasing mitochondrial oxidative stress in skeletal muscle and temporarily reducing mitochondrial membrane potential. A single acute downhill running session significantly activates the PINK1/Parkin pathway in the rat soleus muscle, shown by increased PINK1, Parkin, ubiquitin (Ub), p62, and LC3 protein levels and numerous mitophagosome formations [94]. This study further confirmed through an immunofluorescence double-labeling technique that after exercise, Parkin, Ub, p62 and LC3 co-localized with mitochondria, indicating that acute exercise can rapidly initiate the mitochondrial autophagy program mediated by PINK1/Parkin. Exercise-induced mitochondrial damage was concomitant with the upregulation of PINK1 and Parkin. A study found that after 4 months of aerobic exercise (AE), the content of mitochondria in skeletal muscle increased, and the expression levels of mitochondrial autophagy markers Parkin and BCL2L13 were significantly higher than those before the training [95]. Furthermore, a study has shown that 12 weeks of treadmill exercise training can enhance mitochondrial autophagy activity mediated by PINK1/Parkin by upregulating the SIRT1-FOXO1/3 axis, thereby improving mitochondrial function [96]. These studies collectively demonstrate that exercise effectively eliminates damaged mitochondria and prevents abnormal mitochondria by acutely activating and chronically upregulating PINK1/Parkin pathway, thereby maintaining skeletal muscle.

**Figure 4:**
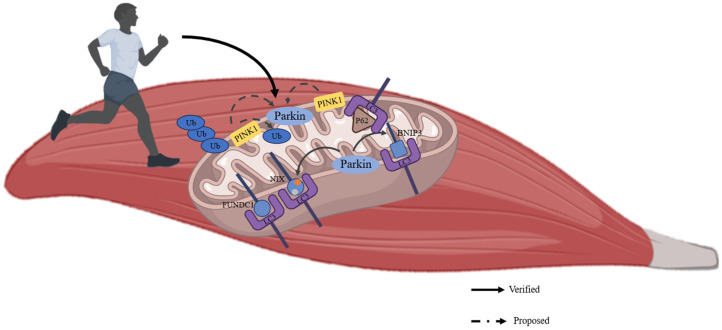
Exercise can activate the PINK1/Parkin pathway, resulting in increased PINK1, Parkin, Ub, p62 and LC3, as well as mitochondrial autophagosomes. On the other hand, exercise can regulate receptor-dependent pathways. Exercise-induced mild hypoxia can upregulate the expression of BNIP3, promoting its binding to LC3 and targeted degradation of damaged mitochondria. Long-term training can reverse the decline in BNIP3 caused by aging, restore mitochondrial autophagy function, prevent abnormal accumulation of mitochondria, and maintain skeletal muscle.

Exercise can also regulate the receptor-dependent mitochondrial autophagy pathway. BNIP3, as an outer mitochondrial membrane protein, can directly bind to the key autophagy protein LC3, thereby directing damaged mitochondria to autophagosomes for degradation [97]. This process is particularly pronounced under the stimulation of exercise, especially during high-intensity exercise when the body is in a state of mild hypoxia, which activates Hypoxia-inducible factor 1-alpha (HIF-1α) [98]. As a transcription factor, HIF-1α can directly upregulate BNIP3, thereby promoting mitochondrial quality under hypoxic conditions [99]. A study has shown that the significant decline in BNIP3 in aged skeletal muscles is intimately linked to mitochondrial impairment and muscular atrophy [100]. However, the decline in BNIP3 induced by aging can be effectively reversed by long-term exercise training, accompanied by a decrease in LC3-II levels. BNIP3-mediated mitophagy can be restored by exercise, with damaged mitochondria being cleared and mitochondrial function reinstated [101] (Figure 4).

## 5. Effects of Different Exercise Patterns on MQC

In AE, the increase in calcium ion concentration caused by muscle contraction and the energy metabolic stress will activate AMPK and further upregulate PGC-1α [102]. As the main regulatory factor for mitochondrial biogenesis, PGC-1α can activate NRFs and Transcription Factor A Mitochondrial (TFAM), thereby initiating the transcription of nuclear genes encoding mitochondrial proteins and mitochondrial genes, ultimately promoting the formation of new mitochondria [103]. The research conducted by Ryotaro Kano et al. [104] indicates that 4 weeks AE can increase mitochondria density in skeletal muscles of mice, while enhancing Citrate synthase (CS) and 3-hydroxyacyl-CoA dehydrogenase (β-HAD), thereby improving the AE metabolic capacity of the body. An animal study conducted by Shota Inoue et al. demonstrated that in middle-aged or elderly mice, after 8 weeks of AE or resistance exercise (RT), PGC-1α in skeletal muscle significantly increased, the rate of protein synthesis increased, and muscle atrophy was reversed [105]. A clinical study conducted by Mary O’Leary et al. further confirmed that 8-week RE could upregulate the expression of PGC-1α, NFF1 and TFAM in the skeletal muscles of middle-aged and elderly subjects and improve body composition and leg muscle strength [106]. Morgane Pengam et al. revealed that compared to moderate-intensity continuous intermittent exercise or resistance training, 6 weeks of HIIT could significantly increase PGC-1α and reduce muscle atrophy [107]. HIIT can activate AMPK/ULK1 through a more intense energy stress. This pathway can directly phosphorylate ULK1 and initiate the autophagy process, thereby more efficiently removing damaged mitochondria [108]. In terms of improving mitochondrial dynamics, a study on a rat model of non-alcoholic fatty liver disease showed that endurance exercise could significantly increase Mfn1, Mfn2, and Opa1 [109]. Another study conducted also found that after 8 weeks of endurance training, mRNA expressions of Mfn1, Mfn2 and Opa1 increased and were associated via mitochondrial network reorganization and the enhanced anti-fatigue ability of the motor units [110]. In a diabetic rat model, it was also found that endurance training significantly increased Mfn2 in skeletal muscle while reducing the fission protein Drp1. This suggests that exercise regulates the balance between mitochondrial fusion and fission [111]. Liang et al. found that 14 weeks of AE could increase DRP1 and FIS1 in skeletal muscles, as well as decrease MFN1/2 and OPA1, improve mitochondrial dynamics, and reduce protein degradation and cell apoptosis, thereby alleviating age-related muscle atrophy [112]. Among the elderly population, 12 weeks of HIIT can significantly increase OPA1 and reduce division protein FIS1, thereby increasing the OPA1/FIS1 ratio. The change in the OPA1/FIS1 ratio is positively correlated with the improvement of mitochondrial respiratory capacity, insulin sensitivity, and cardio-pulmonary fitness [113]. The activation of mitochondrial autophagy by AE mainly occurs by enhancing the PINK1/Parkin pathway, thereby eliminating damaged mitochondria. A 6-week study on aged mice found that AE activates PINK1/Parkin-mediated mitophagy in skeletal muscles, with mice showing increased muscle mass and strength [114]. Sixteen weeks of swimming or rotational exercise can not only increase PINK1 and Parkin but also enhance Bnip3 and alleviate the characteristics of age-related muscle atrophy [115]. Under conditions of aging or pathology, the mitochondrial autophagy function has been impaired. A simple AE intervention is insufficient to reverse this impairment [116]. At this point, it is necessary to combine it with intense exercise stimulation to more effectively activate the autophagy pathway and restore the mitochondrial quality control function [117]. A 5-day study showed that short-term RT failed to alter DRP1 and MFN2 in skeletal muscles of elderly subjects, indicating that RT requires more time to modulate mitochondrial dynamics in skeletal muscles [118]. Paulo HC Mesquita’s research involved 10 weeks RT for the elderly. After 10 weeks, DRP1 and MFN2 in leg muscles of the elderly significantly increased [119]. Additionally, this study confirmed that 10 weeks of RT not only increases mitochondrial density but also modulates markers of mitochondrial dynamics. Effects of different exercise patterns on MQC are detailed in Table 1. ↑ means increase, ↓ means decrease.

## 6. Conclusions

The maintenance of skeletal muscle homeostasis is highly dependent on MQC. The three major processes of mitochondrial biogenesis, dynamic balance, and autophagic clearance are coordinated with each other to jointly ensure mitochondrial network function. MQC imbalance is influenced by multiple factors such as aging, iron homeostasis disorders, energy metabolism disorders, and dysfunction of the autophagic lysosomal system. These factors interweave and jointly disrupt mitochondrial biogenesis, dynamic balance, and autophagic clearance, ultimately leading to skeletal muscle damage. Exercise, as a physiological stimulus, can regulate mitochondrial quality control by activating multiple signaling pathways and remodeling skeletal muscle.

This article provides a systematic review of molecular mechanisms involved in MQC and its impact on skeletal muscle. This article also details how exercise affects mitochondrial quality and examines how different exercise patterns remodel skeletal muscle homeostasis. However, there is still a certain gap regarding whether exercise also regulates skeletal muscle MQC through other pathways and related signaling pathways. Further expansion is needed. Although exercise regulates MQC and shows positive effects in improving skeletal muscle function, whether excessive exercise or excessive activation of MQC is beneficial in all contexts of skeletal muscle still lacks systematic evidence. Therefore, future research should further explore the dose and effect relationship of exercise regulating MQC and the optimal combination of exercise patterns to provide a scientific basis for formulating precise and safe exercise intervention strategies, avoiding shifting from protection to potential damage. While HIIT has been shown to activate mitophagy more efficiently than moderate-intensity continuous training, the threshold intensity, optimal work-to-rest ratio, and minimal effective dose for initiating mitochondrial quality control programs in different populations remain largely undefined. Well-controlled dose-escalation studies integrating molecular readouts of mitochondrial biogenesis, dynamics and mitophagic flux are needed to establish evidence-based exercise prescription for MQC optimization. Future research can also incorporate multi-omics combined analysis techniques to conduct animal and human experiments throughout the life cycle, exploring the mechanism of exercise regulating mitochondrial quality control at multiple systems and levels and clarifying the complete action pathway at the molecular level. Future research should also utilize multi-omics integration methods, combining transcriptomics, proteomics, metabolomics and epigenomics in longitudinal human cohorts and animal models, to comprehensively analyze the complete regulatory network of mitochondrial quality control reshaped by exercise at multiple molecular levels. Single-cell and spatial omics techniques can further elucidate the MQC responses of different cell types to different exercise modalities. This will help identify the key molecular nodes that drive the benefits of exercise-mediated MQC and provide support for the design of precise and individualized exercise interventions throughout the entire life cycle.

## Figures and Tables

**Table 1:** The influence of different exercise patterns on MQC.

Exercise	Subjects	Protein	Functional Changes	Ref.
4-week AE	Male C57BL/6 mice	PGC-1α ↑ CS ↑ β-HAD ↑	Increase mitochondrial density Improve aerobic metabolism capacity	[88]
6-week AE	Aged mice	PINK1 ↑ Parkin ↑	Increase muscle mass Enhance muscle strength	[98]
8-week AE	Aging rats	Mfn1 ↑ Mfn2 ↑ OPA1 ↑ mRNA ↑	Promote mitochondrial network Enhance fatigue resistance	[94]
14-week AE	Aged mice	DRP1 ↑ FIS1 ↑ Mfn1/2 ↓ OPA1 ↓	Improve mitochondrial dynamics Reduce protein degradation Reduce cell apoptosis Alleviate muscle atrophy	[96]
16-week AE	Aging rats	PINK1 ↑ Parkin ↑ Bnip3 ↑	Alleviate muscle atrophy	[99]
6-week HIIT	Male Wistar rats	PGC-1α ↑	Alleviate muscle atrophy	[91]
6-week HIIT	NASH rats	Mfn1/2 ↑	Promote mitochondrial network Enhance fatigue resistance	[93]
12-week HIIT	Old people	OPA1 ↑ FIS1 ↓	Improve mitochondrial respiration	[97]
5 months HIIT	Aging rats	Mfn2 ↑ Drp1 ↓	Optimize balance between mitochondrial fusion and fission	[95]
8-week AE or RT	Aged mice	PGC-1α ↑	Increase protein synthesis Alleviate muscle atrophy	[89]
8-week RT	Older women	PGC-1α ↑ NFF1 ↑ TFAM ↑	Improve body composition Enhance leg strength	[90]
5-day RT	Old people	No significant improvement		[102]
10-week RT	Old people	DRP1 ↑ Mfn2 ↑	Improve mitochondrial dynamics Eliminate damaged mitochondria Increase muscle strength	[103]

## Data Availability

No new data were created or analyzed in this study. Data sharing is not applicable to this article.

## Abbreviations

The following abbreviations are used in this manuscript:

MQC	Mitochondrial quality control
PGC-1	Peroxisome proliferators-activated receptors γ coactivator-1
PRC	PGC-1-related coactivator
PGC-1α	Peroxisome proliIerators-activated receptor γ coactivator lalpha
PGC-1β	Peroxisome proliferator activated receptor gamma coactivator 1beta
ATP	Adenosine Triphosphate
ADP	Adenosine diphosphate
AMP	Adenosine monophosphate
AMPK	AMP-activated protein kinase
SIRT1	Silent information regulator 1
NRF-1	Nuclear respiratory factor 1
NRF-2	Nuclear respiratory factor 2
PINK1	PTEN induced kinase 1
ROS	Reactive oxygen species
OPTN	Optineurin
NDP52	CALCOCO2
P62	Sequestosome-1
LIR	LC3-interacting region
LC3	Microtubule-associated protein 1 light chain 3
FUNDC1	FUN14 domain-containing protein 1
BNIP3	Bcl-2/adenovirus E1B 19-kDa interacting protein 3
NIX	NIP3-like protein X
MFN1	Mitofusin 1
MFN2	Mitofusin 2
OPA1	OPA1 mitochondrial dynamin-like GTPase
DRP1	Dynamin-related protein 1
FIS1	Mitochondrial Fission 1 Protein
FNIP1	Folliculin Interacting Protein 1
ACC	Acetyl-CoA Carboxylase
CaMK	Ca^2+^/calmodulin-dependent protein kinases
MOTS-c	Mitochondrial Open Reading Frame of the 12S rRNA-c
Ub	ubiquitin
HIF-1α	Hypoxia-inducible factor 1-alpha
TFAM	Transcription Factor A Mitochondrial
CS	Citrate synthase
β-HAD	3-hydroxyacyl-CoA dehydrogenase
AE	Aerobic exercise
RE	Resistance exercise
HIIT	High-intensity interval training
